# Bacteriophages as Therapeutic Agents for Pulmonary Infections: From Biological Principles to Clinical Applications

**DOI:** 10.3390/pharmaceutics18030387

**Published:** 2026-03-20

**Authors:** Abdullah A. Alshehri, Alhassan H. Aodah, Ibrahim A. Alradwan, Meshal K. Alnefaie, Majed S. Nassar, Ibtihal S. Alduhaymi, Ahmad M. Aldossary, Nojoud Al Fayez, Essam A. Tawfik, Fahad A. Almughem

**Affiliations:** 1 Advanced Diagnostics and Therapeutics Institute, Health Sector, King Abdulaziz City for Science and Technology (KACST), Riyadh 11442, Saudi Arabia; abdualshehri@kacst.gov.sa (A.A.A.); aaodah@kacst.gov.sa (A.H.A.); ialradwan@kacst.gov.sa (I.A.A.); malnefaie@kacst.gov.sa (M.K.A.); mnassar@kacst.gov.sa (M.S.N.); ialduhaymi@kacst.gov.sa (I.S.A.); nalfayez@kacst.gov.sa (N.A.F.); 2 Wellness and Preventative Medicine Institute, Health Sector, King Abdulaziz City for Science and Technology (KACST), Riyadh 11442, Saudi Arabia; aaldossary@kacst.gov.sa

**Keywords:** bacteriophage, respiratory infection, antibiotic, multidrug-resistant, lung

## Abstract

Respiratory infections remain a significant global health concern, especially as multidrug-resistant (MDR) respiratory pathogens reduce the effectiveness of conventional antibiotics. Patients with chronic lung diseases face persistent biofilm-related infections that are difficult to treat, underscoring the urgency for new solutions. This challenge has renewed focus on bacteriophage therapy as a promising alternative in respiratory antimicrobial management. Bacteriophages are viruses that selectively infect and lyse bacteria, showing strong potential as a precise and effective therapy for resistant pulmonary infections. This review focuses on the mechanisms of phage biology and therapy in lung infections, highlighting their unique interactions with mucus, surfactants, and immune defenses—all of which are central to their clinical promise. The review examines advances in phage engineering, delivery strategies, and inhaled formulations aimed at maximizing phage stability and targeting within the airways. It summarizes recent preclinical and clinical progress targeting MDR respiratory pathogens and discusses regulatory, manufacturing, and safety considerations key to integrating phage therapy into mainstream respiratory care.

## 1. Introduction

Multidrug-resistant (MDR) respiratory pathogens represent a growing global health challenge, largely driven by the overuse and misuse of antibiotics. The rising prevalence of antibiotic resistance has significantly reduced the effectiveness of conventional therapies, making respiratory infections such as pneumonia and tuberculosis increasingly difficult to treat, with some pathogens now resistant to multiple antibiotic classes [1]. In 2024, the World Health Organization (WHO) updated its 2017 Bacterial Priority Pathogens List (BPPL) to address the escalating global threat of antimicrobial resistance more effectively. The revised list ranks 24 antibiotic-resistant bacterial pathogens into critical, high, and medium priority categories. It includes highly resistant Gram-negative bacteria, drug-resistant *Mycobacterium tuberculosis*, and major global threats such as *Salmonella*, *Shigella*, *Neisseria gonorrhoeae*, *Pseudomonas aeruginosa* (*P. aeruginosa*), and *Staphylococcus aureus*. The prioritization is based on global disease burden, transmissibility, limited therapeutic options, and emerging resistance trends. This updated BPPL is intended to guide research priorities, antimicrobial development, investment strategies, and policy actions, with an increased emphasis on targeted, region-specific approaches to mitigate antimicrobial resistance [2]. For example, *Carbapenem-resistant Klebsiella pneumoniae* is identified as the highest-priority threat, with a risk score of 84% due to its resistance to last-resort carbapenem antibiotics, making infections caused by this bacterium particularly difficult to manage [3]. Additionally, Methicillin-resistant *Staphylococcus aureus* (MRSA) remains classified as a high-priority pathogen. However, some healthcare systems have observed declines in MRSA infections due to enhanced infection-control practices and surveillance [3,4].

One advanced approach to target and eliminate MDR respiratory pathogens is bacteriophage therapy. Bacteriophages, or phages, are viruses that specifically infect bacteria and can eliminate them either by multiplying inside the cell until it ruptures in the lytic cycle of bacteriophage or by integrating into the bacterial genome and remaining dormant for several generations before becoming active again, which is called the lysogenic cycle [5]. Bacteriophages were discovered by Frederick William Twort in 1915 and Félix d’Hérelle in 1917, with early observations likely involving phages targeting *Staphylococcus* species that were unintentionally present in laboratory cultures [6].

Phage therapy is being explored in pulmonary medicine because of rising antimicrobial resistance, chronic lung infections, and the limited effectiveness of existing antibiotics, which have made many respiratory infections difficult or impossible to treat [1]. Patients with conditions such as cystic fibrosis (CF), COPD, bronchiectasis, and lung transplants are especially vulnerable to MDR infections, where repeated antibiotic use often worsens resistance rather than resolving the disease. With few new antibiotics in development, bacteriophages offer a promising alternative or additional therapy [1]. Bacteriophages can penetrate and disrupt bacterial biofilms in the airways, which are highly resistant to antibiotics. Topka-Bielecka et al. (2021) [7] reported that the dense extracellular polymeric substance (EPS) matrix of biofilms acts as a physical and chemical barrier that limits antibiotic penetration. In *P. aeruginosa*, exopolysaccharides can bind positively charged aminoglycosides, reducing their diffusion and contributing to antibiotic resistance [7]. In contrast, phages produce enzymes that degrade biofilm structures and bacterial cell walls, facilitating bacterial killing and improving antibiotic penetration [8].

In addition to directly killing pathogenic bacteria, phages can modulate the immune response by reducing excessive inflammation and promoting immune balance, thereby enabling infection control with less lung tissue damage. For instance, it was demonstrated that *Pseudomonas* phage PNM can downregulate Toll-like receptor 4 (TLR4) during phage infection of *P. aeruginosa* [9]. TLR4 is a key pattern-recognition receptor in the innate immune system that detects lipopolysaccharide (LPS) from Gram-negative bacteria and triggers strong inflammatory signaling. Its activation typically triggers the release of pro-inflammatory cytokines and chemokines, which amplify the immune response. Therefore, TLR4 downregulation by the *Pseudomonas* phage PNM can limit excessive inflammation during infection with *P. aeruginosa* [9].

Phage therapy also works synergistically with antibiotics. When bacteria evolve resistance to phages, they may become more sensitive to antibiotics again, reducing overall resistance pressure [10]. Clinical and preclinical studies report high rates of improvement in respiratory infections, including significant bacterial clearance and reduced lung inflammation in MDR pneumonia [11]. Inhaled delivery by nebulization further strengthens this approach by delivering high phage concentrations directly to the lungs, maximizing local effectiveness while limiting systemic exposure [11].

This review aims to comprehensively examine the potential of bacteriophage therapy as an alternative or complementary approach for treating pulmonary infections amid escalating antimicrobial resistance. The scope consists of the biology of bacteriophages, their mechanisms of action against major MDR respiratory pathogens, advances in phage engineering and formulation, and strategies for targeted pulmonary delivery. In addition, the review evaluates current preclinical and clinical studies, addresses regulatory and safety considerations, and discusses key challenges and future directions for integrating phage therapy in the field of respiratory pathogens.

## 2. Biology of Bacteriophages

Bacteriophages exhibit remarkable specificity by targeting particular bacterial species or strains through the recognition of defined bacterial surface receptors [12]. They play a critical role in shaping bacterial population dynamics and enhancing genetic diversity. Their host specificity, biodiversity, and capacity for adaptive evolution highlight the increased interest in using them as therapeutic agents [13]. To develop effective phage-based therapeutics, it is imperative to gain a comprehensive understanding of phage structure, life cycles, and molecular interactions with bacterial hosts. This foundational knowledge will facilitate the advancement of innovative treatment strategies in this emerging field.

Most bacteriophages used therapeutically against respiratory pathogens belong to the order *Caudovirales*. These phages possess double-stranded DNA (dsDNA) enclosed within an icosahedral protein capsid and a tail structure that mediates host attachment and genome delivery. Based on tail morphology and infection dynamics, *Caudovirales* are classified into three main families: *Myoviridae* (contractile tails), *Siphoviridae* (long, non-contractile tails), and *Podoviridae* (short tails) [14].

**Figure 1:**
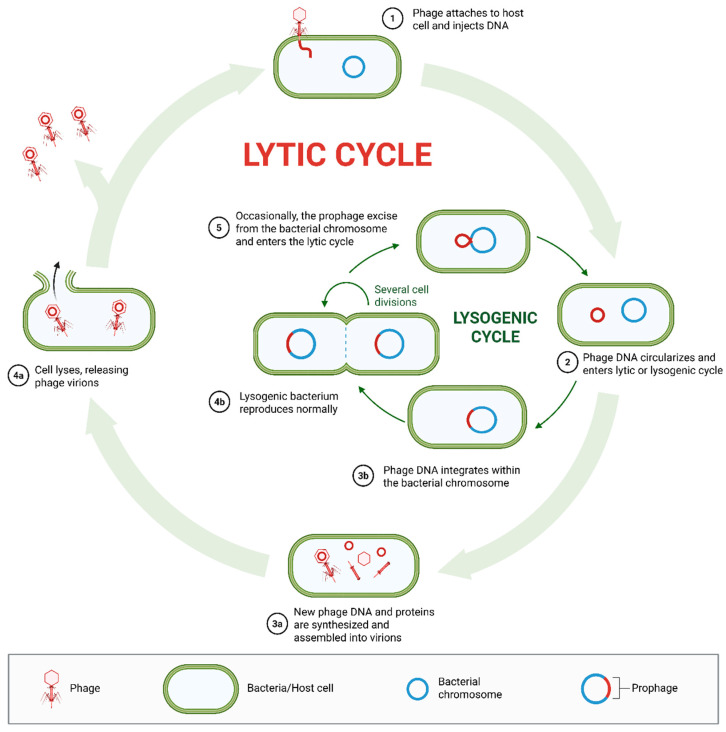
Mechanistic overview of lytic and lysogenic bacteriophage infection cycles in pulmonary bacterial pathogens. Created in BioRender. Al Fayez, N. (2026) https://BioRender.com/1gsp4r2 (accessed on 15 February 2026).

Phages primarily follow two major replication cycles: the lytic and lysogenic cycles [15], as presented in Figure 1. In the lytic cycle, the phage adsorbs to the host cell surface and ejects its genome. It then harnesses the bacterial metabolic machinery to replicate viral components, assemble new virions, and lyse the host cell using phage-encoded endolysins and holins. In this final process, progeny virions are released, typically 50 to 200 per infected cell. This cycle is particularly important and central to therapeutic applications, as it directly eliminates the host bacterium [12]. In contrast, temperate phages alternatively undergo the lysogenic cycle. In this phase, they integrate their genome into the bacterial chromosome as a prophage or persist as plasmid-like episomes. During lysogeny, the phage replicates passively with the host. However, induction conditions, such as host cell stress or DNA damage, can trigger lysis, leading to the release of new virions [16].

Lytic phages are preferable for therapeutic use due to their rapid bactericidal activity and their ability to avoid genomic integration into their host chromosome [17]. While temperate phages contribute to horizontal gene transfer through lysogeny and may increase bacterial virulence, they are generally avoided in therapeutic applications due to the risk of lysogenic conversion and the potential transduction of antibiotic resistance or virulence genes [18]. Therefore, thorough biological characterization and genome screening are essential for selecting therapeutic phages.

Tailed morphologies, dsDNA, and lytic lifecycles are predominantly observed in phages targeting clinically relevant respiratory bacterial pathogens across multiple strains, including *P. aeruginosa*, *Staphylococcus aureus*, *Klebsiella pneumoniae*, the *Burkholderia cepacia complex*, and *Mycobacterium tuberculosis*. Their burst sizes and latent periods are adapted to their hosts’ physiology, reflecting their evolutionary success in complex host environments [19,20,21].

Host–phage interactions are initiated with their adsorption, where phage receptor-binding proteins (e.g., tail fibers) recognize the specific host bacterial surface structures, such as LPS in Gram-negatives, teichoic acids in Gram-positives, pili, and outer membrane proteins [15,22]. These interactions determine host range and infection efficiency, which are critical parameters in respiratory infections characterized by dense mucus layers and heterogeneous bacterial populations.

Phage adsorption is mediated by receptor-binding proteins (RBPs) located on the tail fibers or baseplates of the phage [23]. These proteins bind to receptors on the bacterial surface with high affinity, after which the phage genome is ejected through penetration of the tail tube. This process is supported by virion-associated lysins that degrade peptidoglycan [22]. Within the pulmonary environment, this process occurs in mucus-rich airways that contain surfactants, immunoglobulins, and antimicrobial peptides, all of which can influence phage diffusion and stability. In 2013, a study by Barr et al. indicated that some phages have immunoglobulin-like (Ig-like) domains that promote adherence to mucosal surfaces, thereby enhancing their persistence and antibacterial activity in respiratory tissues [24]. Additionally, many phages produce depolymerases that degrade extracellular polymeric substances, which improves their access to bacterial targets [25].

After adsorption, the ejected genome initiates a tightly regulated replication process that includes early gene expression (host shutdown and DNA replication) and late gene expression (assembly of capsids and tails), followed by packaging. Although phage replication relies on the host’s transcriptional and translational machinery, many therapeutic phages carry auxiliary metabolic genes that optimize replication under stressed and nutrient-limited conditions typical of infected lung tissue [16]. Bacterial host lysis by phages requires holins, pore-forming proteins that permeabilize the cytoplasmic membrane, and endolysins, peptidoglycan hydrolases that enzymatically degrade peptidoglycan. In hypoxic lungs, oxygen-limited alveolar environments, replication may slow, but self-amplifying phages remain effective at infection sites [12,26]. In Gram-negative bacterial pathogens, spanin complexes disrupt the outer membrane, allowing progeny virions to escape. The efficiency of lysis directly affects the phage burst size and therapeutic potency. Phage-encoded depolymerases and biofilm-degrading enzymes significantly enhance penetration into airway biofilms, which are typical of chronic lung infections [27].

For instance, *P. aeruginosa* is a dominant pathogen in chronic infections associated with CF and ventilator-associated pneumonia (VAP). This bacterium is known for forming mucoid biofilms. Phages typically adsorb to *P. aeruginosa* through its LPS O-antigens, type IV pili, or outer membrane proteins. Lytic phages capable of disrupting the bacterial mucoid biofilms by using depolymerases that degrade alginate [28]. Notably, mutations in receptors that confer phage resistance often reduce bacterial virulence or increase susceptibility to antibiotics. This highlights one of the evolutionary trade-offs that is beneficial for therapeutic strategies [29,30]. In addition, phages of the *Myoviridae* and *Siphoviridae* families typically recognize cell wall teichoic acids or surface proteins associated with the MRSA cell wall peptidoglycan. These are the predominant phage families that achieve efficient lysis in both planktonic and biofilm states [31]. In addition to *Staphylococcus aureus*, *Streptococcus pneumoniae* represents another significant Gram-positive respiratory pathogen responsible for community-acquired pneumonia and severe lower respiratory tract infections globally. Similar to other Gram-positive bacteria, pneumococcal bacteriophages typically recognize cell wall-associated structures during adsorption. Several pneumococcal phages exhibit potent lytic activity and encode phage-derived endolysins that degrade the thick peptidoglycan layer characteristic of Gram-positive bacteria. These endolysins have garnered considerable interest as potential antimicrobial agents due to their rapid bacteriolytic activity, specificity toward pneumococci, and their capacity to circumvent traditional antibiotic resistance mechanisms [32]. In addition, phage-derived lysins may contribute to the disruption of bacterial aggregates and early biofilm structures within the respiratory tract, thereby enhancing bacterial clearance in pulmonary infections. However, phages targeting *hypervirulent carbapenem-resistant Klebsiella pneumoniae* (CR-hvKP), which can cause severe pneumonia, often recognize capsular polysaccharides (K antigens) and encode polysaccharide depolymerases that degrade the capsule and facilitate viral adsorption [33]. This degradation of the bacterial capsule, a crucial virulence factor, reduces immune evasion and enhances host immune recognition and clearance in lung tissue [34].

Phages target the *Burkholderia cepacia complex*, which is characterized by its intrinsic resistance group and its role in severe CF infections, typically attaching to its surface polysaccharides or pili. Although these phages have narrow host ranges, they exhibit potent lytic activity against biofilms [35]. Mycobacteriophages, which infect *Mycobacterium tuberculosis*, target slow-growing bacteria with mycolic acid-rich envelopes. Phages such as DS6A and D29 exhibit exceptional genetic diversity and complex life cycles. They penetrate host cells through glycolipids, peptidoglycolipids, or general mycoside C receptors [36]. These host-specific interactions highlight the importance of selecting tailored phages for effective pulmonary treatments.

As with antibiotics, bacteria can develop resistance to bacteriophages through various mechanisms. These include mutations in receptor sites, restriction-modification systems, CRISPR-Cas adaptive immunity, extracellular matrix production, mechanisms that limit phage adsorption, and abortive infection (Abi) systems [37]. In the lung microenvironment, several factors such as immune responses, oxygen gradients, and biofilm formation can accelerate the coevolution of phages and bacteria [38]. In the lung airways, thick mucus can hinder phage diffusion, while biofilms protect inner bacterial cells. Conditions such as hypoxia and mucin can further accelerate alginate production, as observed in *P. aeruginosa* isolated from patients with CF [39]. This helps protect the bacteria from therapeutic phages, antibiotics, and the human immune response. Despite the development of phage resistance, there are often associated costs, such as virulence reduction, impaired biofilm formation, or increased susceptibility to host immunity and antibiotics. This will highlight the strategic potential and effectiveness of using phage cocktails targeting multiple host receptors, or combinations of phages with antibiotics, to prevent the emergence of any resistance [40,41]. Moreover, continuous adaptation of phages, either naturally or through engineering, provides a dynamic advantage over static antimicrobial agents. Understanding these biological principles is essential for designing effective and sustainable phage therapies tailored to the complex, challenging environment of infected lungs.

## 3. Principles and Mechanisms of Phage Therapy in Lung Infections

Although conventional antibiotics remain essential to infection management, their efficacy is increasingly limited in biofilm-associated infections and in the context of MDR pathogens [42]. Limited diffusion through viscous sputum and dense biofilm matrices often results in sub-therapeutic antibiotic concentrations within deeper biofilm layers, promoting bacterial persistence and resistance development. In contrast to broad-spectrum antibiotics that may disrupt commensal microbiota, bacteriophages exhibit high specificity toward their bacterial hosts and typically spare beneficial microbial communities [43]. The absence of a self-amplifying mechanism in antibiotics also necessitates repeated systemic dosing, which may increase toxicity and accelerate antimicrobial resistance [42,44]. In addition, they lack specificity and act broadly, disrupting the normal microbiota and leading to unwanted side effects such as secondary infections. By contrast, therapeutic bacteriophages often exhibit a relatively narrow host ranges, which reduce collateral damage to commensal microbiota [43]. The widespread and prolonged use of antibiotics also poses a major global health challenge by accelerating the emergence of antimicrobial resistance. Finally, because of a lack of an antibiotic self-amplifying mechanism at the site of infection, they require repeated administration at high systemic doses, increasing the risk of allergic reactions and systemic toxicity [42,44]. Collectively, these challenges have driven growing interest in bacteriophage as alternative or complementary antimicrobial agents. These challenges have driven growing interest in bacteriophages as alternative antimicrobial agents, as bacteriophages possess unique biological properties that support their development as antimicrobial therapeutics [45,46]. Therefore, this section summarizes the mechanistic principles underlying phage therapy in the respiratory tract, explains how these mechanisms support its potential application in pulmonary infections, and discusses both potential advantages and key limitations in biofilm-dominated pulmonary infections, such as CF and chronic bronchiectasis.

Phage therapy primarily operates through direct infection and lysis of bacterial cells. At the infection site, phages bind to specific bacterial surface receptors, inject their genetic material into the host cell, and harness host biosynthetic machinery to produce progeny virions. Newly formed phages are then released upon bacterial lysis and can infect neighboring susceptible bacteria, enabling local amplification that reduces bacterial burden, potentially even. This leads to intracellular phage replication and assembly of progeny virions, ultimately causing bacterial lysis and release of new phage particles capable of infecting neighboring bacteria [47,48]. This amplification enables progressive reduction of bacterial burden within infected lung tissues. Unlike conventional antimicrobials, phage replication is driven by the local density of susceptible bacteria and does not depend primarily on dosing and pharmacokinetics. However, phage–bacterium interactions occur within a complex pulmonary environment that influences therapeutic performance. Airway mucus, pulmonary surfactant, and immune responses collectively affect phage stability, distribution, and clearance [45].

Airway mucus is a hydrogel composed mainly of gel-forming mucins such as MUC5AC and MUC5B. These mucins can influence phage retention and distribution within the respiratory tract [49]. Many phages possess immunoglobulin-like domains that facilitate weak interactions with mucins through electrostatic and glycan-mediated binding. This interaction forms the basis of the bacteriophage adherence to mucus (BAM) model [50]. Through BAM, phages accumulate along mucosal surfaces, increasing local phage density and enhancing encounters with invading bacteria. This mechanism may contribute to reduced bacterial colonization of the airway epithelium [24,51]. However, the strength and therapeutic significance of these interactions remain under investigation. In chronic lung diseases such as CF and chronic bronchiectasis, alterations in mucus composition, including altered glycosylation patterns, elevated extracellular DNA content, and increased viscosity, may impede phage diffusion, promote non-productive adsorption, which may yield heterogeneous phage distribution within airway secretions [52]. Consequently, while mucus adherence may enhance phage retention in cerine contexts, the extent to which this mechanism translates to improved therapeutic outcomes in vivo remains unclear and likely varies with phage characteristics and local airway microenvironmental conditions.

This paradigm, however, becomes more complex in the context of chronic respiratory diseases such as CF and bronchiectasis, where pathological remodeling of the airway microenvironment alters the rules of engagement [53]. In these settings, pulmonary pathogens frequently exist not as classic surface-attached biofilms but as suspended, spatially organized aggregates embedded within a dense mucus-biofilm matrix [54]. This pathological architecture can obscure bacterial surface receptors and hinder phage penetration, potentially limiting the efficacy of the BAM model alone [55]. To overcome this, many therapeutic phages have evolved adaptive features that extend beyond simple mucosal adherence. In addition to mucin-binding domains, phages often encode polysaccharide depolymerases, such as alginate lyases, that actively degrade components of both the biofilm extracellular polymeric substance (EPS) and mucin-associated barriers [56,57,58]. This enzymatic activity facilitates deeper penetration into mucus-biofilm complexes, exposes otherwise protected bacterial cells, and creates channels that enhance antibiotic penetration, thereby supporting synergistic phage-antibiotic strategies [58]. Consequently, therapeutic efficacy in the infected lung depends not only on a phage’s lytic capacity but critically on its ability to navigate and disrupt the physical and biochemical obstacles of the mucus-biofilm continuum [59]. Future phage design should therefore prioritize candidates that combine mucus-adhering properties with robust EPS-degrading enzymatic activity, and preclinical models must be refined to more accurately replicate these complex airway environments to improve translational predictability. Such optimizations can be achieved through phage genetic engineering strategies outlined in Section 4, including CRISPR/Cas-mediated payload insertion of depolymerases or synthetic genome assembly, to enhance biofilm disruption and mucus penetration, thereby addressing key limitations in pulmonary applications.

Pulmonary surfactant has been reported to significantly influence the stability, bioavailability, and therapeutic efficacy of phage therapy. It is a complex mixture of phospholipids and surfactant-associated proteins (SP-A, SP-B, SP-C, and SP-D) that collectively regulate alveolar stability and contribute to innate immune defense [60]. Although the hydrophilic surfactant proteins SP-A and SP-D may interact with phage particle surfaces, as with their binding to carbohydrate-rich pathogens, preclinical data indicate that phages retain infectivity against key respiratory pathogens, including *P. aeruginosa*, under physiologically relevant pulmonary surfactant conditions, confirming the feasibility of phage therapy within surfactant-rich distal airspaces [61]. However, their stability and therapeutic performance may depend on capsid characteristics, formulation, and the local inflammatory and immune environment, all of which require careful consideration during the design and development of therapeutic phage preparations intended to reach the distal lung. Moreover, these interactions may influence phage aggregation, diffusion, and clearance, suggesting that the effects of pulmonary surfactant likely depend on phage capsid properties, formulation, and local inflammatory and immune responses. These factors should therefore be carefully considered during the design and development of therapeutic phage preparations intended to reach the distal lung.

It is important to note that following local or inhaled delivery, phages are recognized by the innate immune system via pattern recognition receptors (PRRs) and complement, inducing mild, transient inflammatory responses [62]. Subsequently, they are rapidly cleared by phagocytic cells such as macrophages. With repeated exposure, adaptive immune responses can be activated, leading to production of anti-phage antibodies (mainly IgM and IgG) that neutralize circulating phages and accelerate their clearance [63,64]. Despite this immune recognition, therapeutic phages do not replicate in human cells and typically elicit limited immune activation, with several studies suggesting immunomodulatory effects that help restrain excessive inflammation. At mucosal surfaces, phages can associate with mucus and, through their effects on bacterial communities and epithelial interfaces, indirectly modulate local immune responses, thereby supporting their overall safety in therapeutic and delivery applications [65]. Such immune responses are particularly relevant for chronically infected patients who require repeated dosing or prolonged treatment with phage therapy. For example, a recent clinical report in patients with advanced CF and chronic *P. aeruginosa* infection found that systemic phage therapy, administered in combination with antibiotics, induced phage-specific neutralizing antibodies within 10–15 days of treatment. The emergence of these antibodies was associated with reduced systemic phage activity and coincided with diminished therapeutic efficacy despite early clinical improvement [66]. These findings highlight a potential limitation of repeated phage administration in chronic infections.

Some phages encode depolymerase enzymes that degrade biofilm matrices, enabling deeper penetration and replication at infection sites. Phages can also co-evolve with bacterial hosts, reducing the likelihood of long-term resistance [67]. Together, these properties highlight phage therapy as a targeted, microbiome-sparing alternative to conventional antibiotics. Consistent with this, Zamora et al. reported that phages can directly interact with airway epithelial cells: exposure of human airway epithelial cells to lytic *P. aeruginosa* phages induced transcriptional changes and the secretion of pro-inflammatory cytokines, with the magnitude and nature of these responses varying depending on phage characteristics and the airway microenvironment [68]. These host epithelial effects likely contribute to the heterogeneity of immune reactions during phage therapy. Overall, phage–immune system interactions are complex and may contribute to variability in clinical outcomes, especially in long-standing lung infections with heightened baseline inflammation and complex microbial communities.

A major limitation for antimicrobial therapy in pulmonary infections is the presence of bacterial biofilms. CF and chronic bronchiectasis are characterized by biofilm-dominated bacterial communities embedded within an EPS matrix composed of polysaccharides, extracellular DNA, proteins, and lipids. *P. aeruginosa* lung biofilm, the matrix is particularly rich in exopolysaccharides, alginate, Psl, and Pel. Alginate is a negatively charged polysaccharide associated with the mucoid phenotype frequently observed in CF isolates. Its high viscoelasticity promotes bacterial attachment to surfaces and contributes to protection against phagocytosis and antibiotics [69]. In contrast, Psl and Pel play key structural roles by promoting cell–cell adhesion and maintaining biofilm integrity, thus stabilizing bacterial microcolonies and shielding cells from antimicrobial stress. Together, these EPS components impede the penetration of antimicrobial agents and immune effects, forming mechanically robust and highly hydrated matrices that support bacterial persistence [70].

Many bacteriophages express a polysaccharide depolymerase enzyme that degrades specific components of the EPS matrix and bacterial capsular polysaccharides, thereby improving access to biofilm-embedded cells and potentiating immune and antibiotic activity. These virion-associated enzymes are usually located on tail spikes or fibers and function by enzymatically cleaving extracellular polysaccharides surrounding bacterial cells, thus enhancing access to bacterial hosts and increasing antibiotic penetration [7]. However, the effectiveness of this approach critically depends on the biochemical compatibility between a given phage depolymerase and the target biofilm’s polysaccharide composition.

Although certain phages encode depolymerase enzymes capable of degrading specific EPS components, the matrix can still act as a substantial physical and chemical barrier to phage diffusion when the biofilm composition mismatches the enzymatic specificity of the infecting phage [58]. For example, a recent study characterized *Klebsiella* phage (vB_KpnP_ZX1) encoding a capsule-specific depolymerase (Dep_ZX1) that degrades capsular polysaccharide, reduces biofilm biomass, and enhances immune-mediated bacterial clearance. However, this activity was highly strain-specific, as the phage only infected K57-type *Klebsiella pneumoniae* [71]. Therefore, successful phage therapy in biofilm-dominated lung infections will likely require careful matching of phage enzymatic activity to the biochemical composition of the target biofilm matrix.

The literature describes that bacteria can evolve resistance to bacteriophages through several mechanisms, including receptor modification, restriction-modification systems, and CRISPR-Cas immunity. In response, phages can counter these defenses by switching receptor usage or evading DNA-targeting defense systems [72,73]. In certain environments, this ongoing co-evolution may delay, overcome, or reshape bacterial resistance, leading to stable coexistence rather than permanent phage [74]. Nevertheless, the emergence of resistance remains an important consideration in therapeutic design, and several strategies, such as sequential phage administration, are being developed to broaden bacterial targeting and improve treatment outcomes.

In addition to resistance, the spatial organization of biofilm further influences the phage infection dynamics. For instance, biofilms in the lungs of CF and bronchiectasis exhibit complex spatial structure, consisting of densely packed microcolonies separated by water channels, with gradients in oxygen, nutrients, pH, and metabolic activity [74]. Bacteria near the biofilm surface typically exhibit higher metabolic activity. They are accessible to both antibiotics and phage, whereas cells in the deeper layers often exist in slow-growing or dormant states due to limited oxygen and nutrient availability [75]. Since most lytic phages require metabolically active bacterial hosts for efficient replication, these physiologically inactive subpopulations are less permissive to infection. They can persist as phage-tolerant reservoirs even when superficial layers are effectively targeted [76]. This metabolic heterogeneity represents a major barrier to complete biofilm eradication and contributes to even phage propagation within biofilms, allowing bacterial populations to persist within protected niches despite phage exposure. Recent studies suggest that strategies, including phage-antibiotic combination therapy and depolymerase-expressing targets, could partially overcome metabolic heterogeneity and improve phage efficacy in biofilm-associated infections [77].

Collectively, these observations indicate that although phage therapy offers several potential advantages over conventional antibiotics such as host specificity, localized amplification, and the ability to target certain biofilm-associated infections, its therapeutic effectiveness in pulmonary environments is associate by multiple interacting factors, including mucus composition, immune response, biofilm architecture and phage–host compatibility of conventional antibiotics position phage therapy as a promising, targeted, and microbiome-sparing therapeutic strategy. Table 1 summarizes the key differences between conventional antibiotics and bacteriophages.

## 4. Phage Genetic Engineering and Optimization for Pulmonary Use

Genetic engineering of bacteriophages enables the rational design of therapeutic phages to overcome key challenges in pulmonary delivery. The primary objectives are to enhance phage stability during aerosolization, prolong persistence by reducing immune-mediated clearance, and improve functionality, including efficient penetration of airway mucus and bacterial biofilms, expansion of host range to target MDR strains, and sustained antibacterial activity within the lung microenvironment. These advances are achieved through targeted modification of phage genomes and virion structures using approaches such as payload insertion, receptor-binding protein engineering, and synthetic and systems biology tools. Collectively, these strategies enable the generation of next-generation, optimized phages for the treatment of chronic and drug-resistant respiratory infections.

### 4.1. Classical In Vivo Genetic Engineering

Classical in vivo (host-dependent) genetic engineering relies primarily on homologous recombination within phage-infected bacteria. In this process, genetic modification occurs through recombination between two related phages or between a donor DNA plasmid and the wild-type phage genome during intracellular replication [84]. In addition to natural recombination, random genetic diversity can be introduced into the bacteriophage population via chemical mutagenesis [85], UV exposure [86], or error-prone PCR [87].

When coupled with specific selective pressures, these methods facilitate the isolation of mutants with enhanced or desired functional properties. While this approach provided the foundational proof of concept for early phage engineering, its practical utility, especially for developing therapies against rapidly evolving respiratory pathogens, is hindered by low recombination efficiency and a strict dependency on a viable host for genetic manipulation [88].

### 4.2. Recombineering-Based Phage Engineering

Recombineering-based engineering introduces plasmid-encoded recombination systems, such as λ-Red or RecET, into the bacterial host to increase the efficiency and precision of targeted edits in phage genomes [89,90]. In a relevant pulmonary application, Rebekah and her colleagues genetically engineered three bacteriophages into a cocktail to delete the repressor gene, ensuring the phages were “strictly lytic” and safe for a CF patient with disseminated, drug-resistant *Mycobacterium abscessus*. The phage therapy helped normalize the patient’s inflammatory markers, and lung function was stabilized [19].

### 4.3. CRISPR/Cas-Mediated Phage Genome Editing

The adaptation of CRISPR/Cas systems has revolutionized bacteriophage genome editing by providing three powerful, complementary applications: negative selection against wild-type phages, precise, targeted modification of phage genomes, and phage delivery of CRISPR/Cas payloads to edit bacterial pathogens destructively. These methodologies enable the creation of phages with specifically tailored properties, overcoming the inefficiency and randomness of traditional recombination-based techniques [91,92].

In the first approach, CRISPR/Cas is employed as a selective pressure to eliminate wild-type phage genomes from a mixed population. Host bacteria are engineered to express CRISPR-Cas machinery and guide RNAs (gRNAs) that specifically target the phage genome’s wild-type sequence. Upon infection, the wild-type phages are efficiently cleaved by the Cas nuclease. In contrast, recombinant phages carrying desired genetic modifications, such as point mutations, insertions, or deletions that disrupt CRISPR target-site escape cleavage, are selectively enriched. This robust negative selection dramatically purifies the final phage population for the engineered genotype, effectively filtering out unmodified backgrounds and increasing engineering efficiency. This strategy is particularly valuable for isolating rare homologous recombination events or identifying bacteriophages with specific point mutations from large mutagenic libraries [93].

The second approach is a direct editing strategy in which CRISPR/Cas creates a targeted double-strand break (DSB) in the phage genome to stimulate precise repair. Following Cas-mediated cleavage, the phage genome is deliberately modified through homology-directed repair or recombination. In this context, donor DNA templates encoding the desired genetic changes are provided either on plasmids or as linear DNA fragments [91,92]. This enables precise insertion of functional modules, such as biofilm-degrading enzymes, immune-evasion elements, or engineered receptor-binding proteins. This method facilitates the rational, site-specific modification of phage genomes and supports the modular construction of multifunctional therapeutic phages [94]. An important application of CRISPR/Cas-mediated phage genome editing in pulmonary therapeutics is the targeted modification of phages to express enzymes that disrupt biofilm formation, a major barrier to effective treatment of chronic lung infections. For instance, Luo et al. engineered two *P. aeruginosa* phage variants using CRISPR/Cas9: one to express a quorum-quenching enzyme (AiiA lactonase) to disrupt bacterial cell-to-cell signaling, and the other with a depolymerase (PelA) to degrade the Pel exopolysaccharide in biofilms. Both engineered phages demonstrated significantly enhanced inhibition of biofilm formation and disruption of pre-established biofilms in vitro compared to the wild-type phage, showcasing the utility of precise payload insertion for combating biofilm-associated pulmonary infections [95].

Beyond direct phage genome editing, a third and increasingly important approach uses phagemids to deliver CRISPR/Cas systems as antimicrobial payloads. Phagemids are plasmid-derived vectors packaged into phage capsids that retain phage-mediated delivery capabilities while lacking full phage replication machinery. In this strategy, Phagemids encoding CRISPR/Cas components, typically Cas nucleases and gRNAs targeting antibiotic resistance genes or essential bacterial loci, are delivered into bacterial cells by helper phages. A pioneering example for respiratory applications is the use of the CRISPR/Cas13a system to achieve gene-specific reversal of antibiotic resistance, unlike Cas9, which cleaves DNA, Cas13a targets sequence-specific mRNA transcripts. In a study focusing on carbapenem-resistant *P. aeruginosa*, phagemids were engineered to deliver a CRISPR-Cas13a payload, capped at the P minus 1 end. This approach markedly reduced the minimum inhibitory concentration of imipenem and restored carbapenem susceptibility in clinical *P. aeruginosa* isolates [96].

### 4.4. Synthetic Genome Assembly

Synthetic genome assembly (ex vivo) approaches represent a transformative advancement for engineering bacteriophages tailored to treat pulmonary infections [97]. Unlike traditional intracellular methods, which are hindered by host dependence and low efficiency, these host-independent, cell-free strategies enable precise, modular genome design [98]. The process involves assembling engineered phage genomes in vitro from PCR-amplified fragments using Gibson Assembly or yeast-based platforms, followed by reactivation into infectious particles via electroporation or cell-free transcription-translation systems [99]. Several bacteriophages have been assembled using this approach to target, for example, *P. aeruginosa* [100], *Staphylococcus aureus* [101], and *Escherichia coli* [97]. For pulmonary applications, such synthetic platforms enable rationally engineered phages tailored for inhaled delivery, optimizing stability, capsid chemistry, and broad targeting of respiratory strains, a promising strategy for complex lung infections despite its technical demands.

### 4.5. Non-Genetic Modification

Beyond genetic and synthetic biology-based engineering, non-genetic modification strategies represent a critical complementary pathway for optimizing bacteriophages in pulmonary applications. These approaches are particularly valuable for ensuring phages survive immune surveillance, penetrate mucosal barriers, and withstand the mechanical stresses inherent in aerosol delivery. Furthermore, non-genetic strategies offer distinct advantages regarding regulatory approval pathways and the preservation of the phage’s native biological functionality [101]. For instance, Sawant et al. developed PEG-stabilized liposomes (comprising HSPC, DSPG, DSPE-PEG, and cholesterol) designed to provide both mechanical shielding and controlled release. This formulation enabled sustained release for 10 h and significantly enhanced mechanical stability, reducing nebulization-induced viability loss from 1.55 log to 1.08 log. Additionally, encapsulation achieved a two-fold reduction in cellular uptake, thereby promoting prolonged extracellular retention within in vitro lung epithelial models [102].

## 5. Local Delivery and Formulation Strategies to the Respiratory System

Nebulization is a process that converts liquid solutions into fine aerosols, making it an appropriate method for pulmonary drug delivery. This technique can deliver large volumes while producing particle sizes suitable for deposition in the lower airways [103]. For this reason, nebulization is considered the primary method for delivering phages into the respiratory tract via aerosolization. Several in vitro and in vivo studies have investigated inhaled phage therapy using nebulizers, demonstrating its applicability for respiratory delivery [47].

Nebulizers operate via different mechanisms, including vibrating mesh, compressed-air jet, ultrasound, and colliding liquid jets. Jet nebulizers use compressed gas to produce aerosol, while larger droplets are recycled for re-nebulization. During this process, phages may be exposed to mechanical stress, leading to reduced titer due to repeated impaction against the baffle [104]. Ultrasonic nebulizers generate aerosols through piezoelectric crystals; however, heat generation during operation can degrade phages and proteins, making these systems less suitable for viscous or proteinaceous solutions [103]. Mesh nebulizers include vibrating mesh and static mesh systems. Vibrating mesh nebulizers use vibrating plates with microscopic holes to generate aerosols. They may cause significant phage damage, whereas static mesh nebulizers use ultrasonic vibrations to push liquid through a static mesh to produce aerosols [103].

The structural stability of the anti-*pseudomonal* phage PEV44 has been evaluated after nebulization with air-jet, vibrating-mesh, and static-mesh nebulizers. Jet nebulization was associated with severe structural damage affecting approximately 83% of phage particles, whereas mesh-based nebulizers caused damage to about 50% of the phage population. These observations demonstrate that mechanical stress during nebulization markedly influences phage viability and that the extent of damage is strongly dependent on the type of nebulizer employed [47]. Further studies have reported inconsistent results regarding phage survival during nebulization, with outcomes influenced by phage type and nebulizer model. Jet nebulization can expose phages to repeated mechanical stress, often causing phage damage such as tail detachment or nucleic acid ejection [103,105]. In some cases, vibrating mesh nebulizers have been associated with greater titer reduction compared to jet nebulizers, although these effects vary among different phage types [103].

Several nebulization methods, including atomizer-based systems, multi-jet collision nebulizers, and mesh nebulizers, have been evaluated using structurally diverse bacteriophages (MS2, Φ6, ΦX174, PM2, and PR772). The results demonstrated that phage stability during nebulization is strongly phage-dependent, with susceptibility to nebulization-induced stress varying with phage structure and characteristics [47]. Overall, phage viability during nebulization is influenced by mechanical stress, phage type, nebulizer type, relative humidity, and temperature. Vibrating mesh nebulizers are generally more efficient and less damaging compared to jet nebulizers. Therefore, selecting robust phages and optimizing nebulization conditions are critical to the success of phage therapy via inhalation. Despite existing challenges related to phage stability and nebulizer-specific effects, nebulization remains a promising method for phage therapy, particularly for pulmonary infections, and requires further investigation [47,103].

Dry Powder Inhalers (DPIs) are increasingly explored as pulmonary delivery systems for bacteriophage therapy, particularly for respiratory infections. DPIs are portable, simple to use, and do not require regular cleaning or disinfection, making them an attractive alternative to nebulizers for inhaled bacteriophage administration. Moreover, DPIs do not require electricity, thereby enhancing their applicability for patients in developing regions and facilitating broader access to bacteriophage-based treatments [47]. A key advantage of DPIs for bacteriophage delivery is their ability to deliver high therapeutic loads directly to the lungs with rapid drug administration. Compared to nebulizers or pressurized metered-dose inhalers, DPIs offer improved formulation stability and enable the delivery of higher bacteriophage concentrations, supporting their effectiveness as pulmonary delivery platforms [106]. These characteristics make DPIs particularly suitable for localized treatment of respiratory infections caused by bacterial pathogens [103].

The solid-state nature of DPI formulations enhances the stability of bacteriophages by protecting them from mechanical shear, enzymatic degradation, and environmental stress. Bacteriophage powders formulated for DPIs have been shown to maintain biological activity over years of storage with minimal titer loss of less than 2 log_10_ [103,107]. Consequently, dry powder formulations represent a stable and convenient approach for bacteriophage delivery, offering promising long-term storage capabilities for respiratory applications [103]. Several DPI system designs have been developed to accommodate bacteriophage formulations. Carrier-based DPI systems employ excipients such as lactose or mannitol to enhance powder flow and dispersion; however, these excipients may dilute the bacteriophage payload [105]. In contrast, carrier-free DPI systems eliminate excipients to maximize bacteriophage potency, payload, and aerodynamic performance. Nanoparticle-embedded DPI systems incorporate polymeric or lipid-based carriers to enhance bacteriophage protection, enable controlled release, and improve delivery efficiency [105]. Optimization of particle size (1–5 µm), density, and morphology is critical to ensure efficient lung deposition and to avoid clearance by alveolar macrophages, while carrier-free formulations allow higher bacteriophage payloads and greater flexibility for personalized phage therapy [105].

The method of powder preparation plays a critical role in preserving bacteriophage viability in DPI formulations. Milling has been reported to damage bacteriophages; therefore, lyophilization and spray drying are preferred methods for preparing inhalable bacteriophage powders [108]. These approaches support the production of dry powders suitable for pulmonary delivery while maintaining bacteriophage structural integrity and biological activity [108]. Several powder development techniques are used to prepare bacteriophage DPI formulations, including freeze-drying, spray-drying, and electrospraying. Freeze-drying preserves the highest level of bacteriophage activity, followed by spray-drying, while electrospraying produces powders with the highest bulk and tapped densities. Excipients such as whey protein, inulin, gum arabic, and Tween 80 are commonly used to stabilize bacteriophages during drying. Overall, DPIs offer a promising alternative to nebulized systems for pulmonary bacteriophage delivery by combining stability, efficiency, and ease of use [105].

Compared with nebulized bacteriophage delivery systems, DPIs exhibit lower loss of phage titer. Nebulized *Myoviridae* bacteriophages exhibit losses of 0.65–1.2 log plaque-forming units (PFU), while *Podoviridae* bacteriophages lose 0.3–0.8 log PFU. In contrast, optimized DPI formulations retain up to 90% bacteriophage viability during drying and storage, contributing to improved shelf life, ease of use, and patient compliance compared to liquid aerosol delivery via intranasal instillation [105].

Intranasal bacteriophage therapy is a promising, non-invasive approach for delivering therapeutic agents directly to the lungs and the central nervous system (CNS) to treat localized infections. In early clinical trials, intranasal administration of phage AB-SA01 in patients with chronic rhinosinusitis (CRS) demonstrated safety and tolerability, with no serious adverse events reported even at high concentrations over 14 days. This aligns with studies showing that phage therapies administered via other routes, such as topical or oral delivery, are similarly well tolerated, though further randomized, placebo-controlled trials are required to confirm efficacy and establish standardized safety endpoints [109].

**Figure 2:**
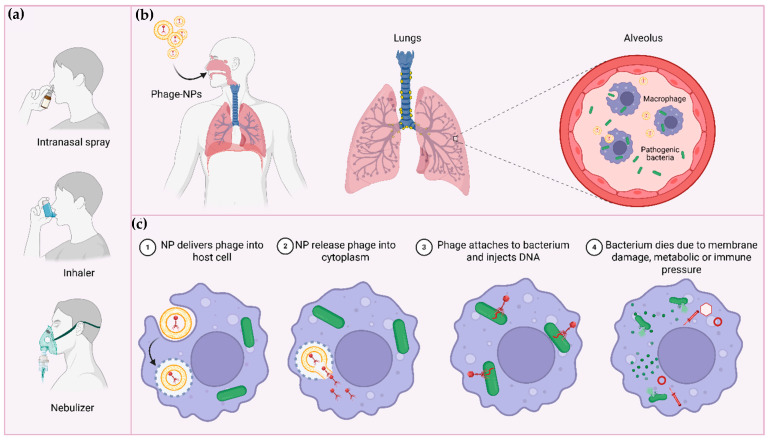
Schematic overview of respiratory tract delivery and intracellular targeting of nanoparticle-encapsulated bacteriophages. (**a**) Administration routes for pulmonary delivery, including intranasal sprays, inhalers, and nebulizers. (**b**) Deposition of administered nanoparticles within the alveolar cavity followed by cellular internalization by alveolar macrophages. (**c**) Intracellular release of bacteriophages and subsequent targeting of intracellular bacteria, leading to phage-mediated bacterial killing via non-productive (abortive) infection mechanisms. Created in BioRender. Al Fayez, N. (2026) https://BioRender.com/mfiz3ze (accessed on 15 February 2026).

Additionally, the intranasal route has been explored to target the CNS via the olfactory system, which facilitates direct transport of therapeutic agents to the brain. For example, filamentous phages displaying anti-β-amyloid antibody fragments were successfully used to label β-amyloid plaques in transgenic mice. These phages specifically targeted plaques in key brain regions, such as the olfactory bulb and hippocampus, while maintaining their biological activity and effectively penetrating biological membranes. Importantly, no phage presence was observed in these regions after systemic administration, emphasizing the efficiency and specificity of the intranasal route for CNS targeting [110]. Figure 2 shows a schematic overview of respiratory tract delivery and intracellular targeting of nanoparticle-encapsulated bacteriophages.

## 6. Biodistribution and Pharmacokinetics of Therapeutic Bacteriophages

Bacteriophages distribute rapidly after systemic administration, with preferential accumulation in the liver and spleen, which function as biological “sinks” because macrophages in the mononuclear phagocyte system take them up. The route of administration and dosage strongly influences circulation time and organ localization, whereas the contribution of intrinsic phage properties, such as size and structural features, remains less clearly defined [111]. Phage absorption following oral or inhalation administration is influenced by phage size, morphology, and the cell types present at the site of administration [112].

Pulmonary biodistribution and pharmacokinetics of inhaled phages are significantly affected by bacterial infection. Inhalation generally results in higher phage recovery in lung tissues than intraperitoneal injection; however, murine models of *Burkholderia cenocepacia* pulmonary infection have demonstrated that intraperitoneal administration can elicit stronger antibacterial activity despite lower phage localization in the lungs [111]. These findings indicate that therapeutic efficacy is not solely determined by local phage concentration but is also influenced by host-pathogen interactions.

Detailed pharmacokinetic evaluation of the antipseudomonal phage PEV31 following pulmonary administration in mice further highlights these dynamics. Intratracheal delivery of doses ranging from 10^7^ to 10^9^ PFU resulted in detectable phage levels in lung tissue for up to 24 h. In the absence of bacterial infection, lung phage titers declined gradually, with an estimated pulmonary half-life of approximately 8 h at both dose levels, underscoring the role of bacterial presence in prolonging phage persistence in lung tissues [113].

Host factors, phage morphology, and immune responses also influence phage pharmacokinetics. Distribution between blood and organs is partly size-dependent, while environmental conditions such as pH and microbiome composition may influence phage stability and clearance [112]. Collectively, these factors contribute to key pharmacokinetic challenges, including achieving effective host penetration, appropriate tissue distribution, and overcoming clearance mediated by immune and physiological responses [114].

In IV administration, the size of bacteriophages plays a critical role in both biodistribution and pharmacokinetics. In terms of biodistribution, phages ranging from 20 to 200 nm are too large to be cleared by the kidneys, which restricts clearance to particles smaller than 8 nm. This leads to their accumulation in the liver and spleen, where the mononuclear phagocyte system (MPS) facilitates clearance. The liver, through Kupffer cells, is more effective at degrading phages, while the spleen retains phages longer, acting as a “phage sink.” Despite this, phages can penetrate blood barriers and distribute to other tissues, including the brain, lungs, and kidneys, with localization influenced by dosage and route of delivery [115].

For pharmacokinetics, larger phages are cleared more rapidly from circulation than smaller ones. For example, the larger T2 phage showed faster clearance in pigs compared to the smaller ΦX174 phage, with only 0.1% of T2 remaining in circulation after 24 h, while over 10% of ΦX174 persisted. While size-dependent clearance is evident, structural features like capsid proteins have minimal impact. Filamentous phages like M13 demonstrate slightly shorter half-lives (~4.5 h) than nonfilamentous phages like lambda (~5–6 h). These findings highlight the significant influence of size on biodistribution and pharmacokinetics in IV administration [115].

PK/PD modeling faces several challenges in accurately replicating biological conditions. In vitro studies do not fully replicate in vivo conditions because they lack factors such as immune system influence, tissue distribution, and bacterial resistance development, which necessitate the use of dynamic PK/PD models to better mimic in vivo environments [114]. Animal models, while bridging the gap between in vitro and clinical studies, face limitations in replicating human immunity and physiology, with differences in dosing, infection progression, and interspecies variations further complicating the translation of findings to clinical applications [114,116]. Additionally, bacteriophage clearance is influenced by immune system interactions, such as uptake by immune cells, making PK modeling more complex. Differences between healthy and infected individuals, particularly due to phage self-replication in the presence of susceptible bacteria, add another layer of difficulty [114,116]. The transient presence of phage-resistant bacteria poses challenges for predicting therapeutic outcomes, and PK/PD models often rely on assumptions that do not fully reflect the complexity of biological environments, leading to discrepancies between model predictions and real-world outcomes. While in vitro models may predict bacterial resistance patterns, they often fail to correlate with clinical outcomes, further highlighting the challenges in PK/PD modeling [114,116].

## 7. Preclinical and Clinical Applications in Pulmonary Diseases

Murine lung infection models remain the cornerstone of preclinical phage development for respiratory indications because they enable precise control of bacterial inoculum, treatment timing, and route of administration, as well as quantitative efficacy and mechanistic readouts [117]. Common endpoints include lung and bronchoalveolar lavage (BAL) bacterial burden measured as colony-forming units (CFU), phage exposure measured as PFU, inflammatory cytokines such as TNF-α (tumor necrosis factor α), IL-6 (interleukin-6), and IL-1β (interleukin-1 β), alongside histopathology and survival [118]. Phages are typically delivered intratracheally for accurate lung dosing, intranasally as a simplified airway proxy, or by nebulization, closely aligning these models with pulmonary translation [119].

Acute murine pneumonia models have been particularly informative for linking pulmonary pharmacokinetics, in vivo phage amplification, bacterial killing dynamics, and resistance emergence [118]. A well-characterized example is the inhaled antipseudomonal phage PEV20 evaluated in neutropenic mice [61]. Following intratracheal administration at 10^7^ to 10^9^ PFU, PEV20 displayed a pulmonary half-life of approximately 8 h in healthy lungs. In infected lungs, phage levels increased by nearly 5.3 log_10_ units within 22 h, consistent with replication at the site of infection [120]. This amplification correlated with suppression of MDR *P. aeruginosa* growth and enabled direct quantification of resistance emergence; a subset of recovered isolates exhibited altered antibiograms and reduced virulence-associated traits [120]. Together, these studies illustrate how acute models can integrate route, dose, lung exposure, efficacy, and resistance within a single experimental framework.

Dose selection has been a major translational gap in inhaled phage therapy, and murine dose-response studies are beginning to address this limitation [121]. In respiratory infection models using PEV31, escalating pulmonary doses were associated with increased frequencies of phage-resistant mutants, reported at approximately 30%, 74%, and 91% across ascending dose groups [121]. Importantly, higher doses were also linked to the suppression of inflammatory mediators, including IL-1β, IL-6, and TNF-α, highlighting a complex trade-off among antibacterial efficacy, host response modulation, and resistance selection [121]. These findings support framing pulmonary phage therapy as a pharmacokinetic and pharmacodynamic optimization problem rather than assuming that higher doses are inherently superior.

Chronic and CF-relevant models extend these insights to settings of biofilm-associated persistence and antibiotic tolerance [122]. Agar-bead lung infection models using mucoid *P. aeruginosa* strains recapitulate key features of chronic colonization. They are particularly valuable for evaluating delayed treatment, repeated dosing strategies, and resistance trajectories under sustained selective pressure [123]. In one representative design, mice infected intratracheally with agar bead-embedded bacteria and treated with a single intratracheal dose of the phage PEV20 achieved multi-log reductions in lung bacterial burden within 24 h. A significant reduction (−5.3 log_10_) in bacterial load was observed in the treated group compared with the nontreated group, with complete clearance by one week and concurrent reductions in pro-inflammatory cytokines [124]. Such models provide a biologically relevant platform for testing phage performance in conditions that more closely resemble chronic airway disease.

Beyond mice, rats are frequently used for aerosolization studies and ventilator-associated paradigms as they better accommodate airway instrumentation and physiologic monitoring [125]. Nebulized phage therapy has been evaluated in rat models of MRSA pneumonia for both prophylaxis and treatment, demonstrating reductions in lung bacterial load and improvements in survival, thereby supporting inhaled delivery strategies [125,126]. More recently, murine VAP models with defined inocula and clinically structured dosing schedules have enabled evaluation of adjunctive phage-antibiotic therapy [127]. In these models, combination treatment with phage cocktails and antibiotics reduced lung and BAL bacterial burdens by 2–3 log units, improved clinical status, and attenuated lung injury compared with either monotherapy [127]. Collectively, animal data across acute, chronic, and ventilator-associated pulmonary models converge on several consistent translational principles. Therapeutic efficacy is most robust when airway delivery achieves sufficient local exposure, dosing is initiated early or repeated appropriately, and phages are combined with antibiotics to enhance killing kinetics and limit resistance selection.

Current human evidence for pulmonary phage therapy remains limited and heterogeneous. Most reported clinical experiences derive from compassionate use programs, expanded access programs, case reports, and small observational cohorts. These studies primarily demonstrate feasibility, microbiological response, and safety rather than definitive clinical efficacy. However, building on the strong preclinical foundation, clinical evidence is gradually expanding but remains uneven across pathogens and study designs [128]. Only a limited number of randomized clinical trials are currently underway, and robust efficacy data from large, well-powered studies are still lacking [128]. Nevertheless, existing reports increasingly include quantitative microbiologic and functional endpoints, helping connect preclinical findings to early clinical translation.

For *P. aeruginosa*, pulmonary phage therapy is the most mature clinically [118]. Early expanded-access cases of chronic MDR lung infection treated with aerosolized phages documented clinically meaningful improvement despite incomplete bacterial eradication, including reductions in sputum bacterial burden and decreased reliance on systemic antibiotics [129]. More recently, a compassionate-use cohort in CF provided greater granularity on the feasibility and heterogeneity of response. In this series of 9 adults with CF, nebulized phage therapy delivered at a total dose of approximately 1 × 10^10^ PFU per administration over 7 to 10 days reduced median sputum *P. aeruginosa* density from 2.6 × 10^8^ to 2.6 × 10^4^ CFU/mL within days of treatment, with sustained suppression at later follow-up [130]. These reports should therefore be interpreted as early clinical observations rather than controlled evidence of efficacy. Lung function also improved modestly, with median percent-predicted FEV1 (forced expiratory volume in one second) increasing from 36% to 42%, and no major safety signals were observed [130]. Importantly, this clinical trajectory now extends into randomized evaluation. The CYPHY Phase 2 trial is a double-blind, placebo-controlled study in CF patients with *P. aeruginosa*, enrolling 36 participants to receive 7 days of inhaled phage therapy [131]. Its primary endpoint focuses on quantitative change in sputum bacterial load, reflecting a shift toward standardized microbiologic efficacy measures aligned with preclinical pharmacodynamic principles [131]. Although several randomized clinical trials are currently underway, large, well-powered studies demonstrating definitive clinical benefit remain limited.

Clinical use of phages in pulmonary infections against other highly resistant Gram-negative pathogens, including *Klebsiella pneumoniae* and *Acinetobacter baumannii*, is increasing but remains largely confined to case reports and small series [132]. These accounts often involve aerosolized phages, sometimes combined with systemic administration, in patients with severe or VAP [132]. The most informative data from these reports are microbiologic rather than clinical, including temporal tracking of phage detection and bacterial burden. In one characterized case report of drug-resistant *Acinetobacter baumannii* pneumonia, inhaled phage therapy was associated with marked increases in sputum phage signal by quantitative PCR, transient low-level systemic detection, and parallel reductions in respiratory bacterial load [133]. Such studies highlight feasibility and biological activity, but also underline variability in routes, dosing, and endpoints, reinforcing the need for harmonized outcome measures such as sputum CFU kinetics, time to culture negativity, ventilator-free days, and inflammatory markers [134].

In contrast, the clinical evidence base for pulmonary phage therapy targeting *Staphylococcus aureus* remains comparatively underdeveloped [135]. Although multiple preclinical pneumonia models support the mechanistic rationale for inhaled phages against MRSA, rigorously controlled human respiratory trials are scarce [135]. The available clinical reports establish feasibility, tolerability, and biological activity, but fall short of demonstrating reproducible therapeutic efficacy [136]. For example, a clinically structured rat model of VAP demonstrated that a single prophylactic dose of nebulized phage cocktail improved survival from 0% in controls to 60–70% in treated animals and reduced lung bacterial burden by approximately 500-fold, with phages largely confined to pulmonary tissue [137]. Despite this strong proof of concept, the study also underlined unresolved questions regarding dosing, distribution, and efficacy in heterogeneously ventilated human lungs [137]. Consequently, current progress in *S. aureus* pulmonary phage therapy remains driven predominantly by preclinical data, highlighting a translational gap and the need for well-designed early-phase clinical trials.

Overall, current human studies suggest that inhaled phage therapy for pulmonary infections is feasible and biologically active, with the strongest evidence to date in *P. aeruginosa*, particularly in CF [105]. However, the field remains constrained by small sample sizes, heterogeneous designs, and limited standardized endpoints. These limitations mirror those identified in preclinical models and reinforce the importance of integrating pharmacokinetics, microbiologic dynamics, and resistance monitoring into future well-powered randomized trials [138].

Following the broader human clinical experience, compassionate use, defined as the regulated clinical administration of investigational therapies outside formal trials for patients with serious or life-threatening disease lacking satisfactory treatment options, has played an important role in advancing phage therapy toward translational relevance. In this context, CF and VAP are two settings in which phage therapy has progressed from proof of concept to clinical application. In CF, compassionate use has served as a critical bridge, reflecting the persistent burden of MDR *P. aeruginosa* and limited therapeutic alternatives [130]. Across reported case series and small cohorts, inhaled phage therapy has generally shown acceptable tolerability with heterogeneous microbiologic responses [130]. Notably, clinical benefits such as reduced exacerbation frequency or stabilization of lung function have sometimes been observed despite incomplete bacterial eradication, reinforcing the concept that symptom control and inflammation modulation may be clinically meaningful outcomes alongside culture negativity.

VAP poses a distinct translational challenge, in which timing, disease severity, and host injury critically influence outcomes [127]. Here, advances have been driven primarily by clinically structured animal models rather than human case series. Recent murine VAP models incorporate ventilator-induced lung injury, standardized bacterial inocula, and timed therapeutic interventions, thereby approximating key elements of intensive care unit physiology [127]. In a representative model of *P. aeruginosa* VAP, mechanically ventilated mice received phage therapy, antibiotics, or a combination of both at defined early and delayed time points. Beyond reductions in lung bacterial burden, efficacy was assessed using clinical disease scores, temperature, and weight changes, and markers of epithelial injury [127]. Combination phage antibiotic therapy consistently produced faster clinical improvement and reduced lung damage compared with monotherapy, while also lowering the effective antibiotic exposure and limiting resistance emergence [127]. Importantly, VAP models explicitly encode variables that clinical studies must define in advance, including treatment timing, dosing frequency, antibiotic partners, and endpoints that extend beyond CFU reduction alone.

As pulmonary phage therapy advances toward structured clinical deployment, the choice between monophage and phage cocktail strategies has emerged as a central design consideration [130]. This decision reflects a balance between potency, breadth of coverage, resistance management, and manufacturing feasibility, and is particularly relevant in CF and other settings characterized by within-host bacterial diversity and evolution [130]. Monophage therapy offers high phage potency when a single lytic phage is carefully matched to a dominant patient isolate [130]. This approach benefits from simpler characterization, more explicit dose definition, and tighter manufacturing control, making it attractive when susceptibility is stable and clonality is high [139]. In a compassionate-use cohort of 9 adults with CF, individualized phage selection led to single-phage therapy in 3 patients at a standardized total dose of 1 × 10^10^ PFU per administration over 7 to 10 days [139]. Despite incomplete eradication, sputum *P. aeruginosa* burden decreased by approximately 4 orders of magnitude, from a median of 2.6 × 10^8^ to 2.6 × 10^4^ CFU/mL shortly after treatment, with parallel improvements in lung function as reflected by an increase in median percent predicted FEV1 from 36 to 42 [139]. In selected cases, monophages such as OMKO1 were also chosen to exploit receptor trade-offs, with post-therapy isolates exhibiting increased susceptibility to multiple antibiotics [29].

Phage cocktails, in contrast, are designed to provide broader host range coverage and to reduce the probability of rapid resistance escape, particularly when constituent phages target distinct bacterial receptors [140]. This can be advantageous in heterogeneous colonization, polymicrobial infection, or empiric deployment where isolate-specific susceptibility is uncertain. Large-scale in vitro screening efforts have demonstrated that rationally designed cocktails can achieve high activity against diverse clinical *P. aeruginosa* isolates (PA14-mCherry, PAO1-GFP, and a clinical isolate of CPA050) [141]. The cocktails were developed using a receptor-guided phage cocktail design in which lytic phages were grouped into complementarity groups based on distinct bacterial surface receptors to maximize host-range coverage and limit resistance. For *P. aeruginosa*, a three-phage cocktail combining Luz24 (type IV pilus-dependent), OMKO1 (flagella and OprM-dependent), and PAML-31-1 (LPS-dependent) achieved suppression of ≥96% of 153 clinical isolates, including biofilm and polyclonal populations, while reducing single-step resistance compared with a monophage [141]. For *Staphylococcus aureus*, a two-phage cocktail targeting different cell wall structures expanded coverage and showed enhanced activity when combined with vancomycin [141].

Similarly, the development of the nebulized three-phage product of the BX004-A clinical trial relied on a CF-derived panel of 143 donor strains [142]. The BX004 is composed of genetically distinct, strictly lytic phages targeting different *P. aeruginosa* surface receptors, collectively lysing 106 of 143 CF clinical isolates while maintaining activity against biofilms and stability during aerosol delivery [142]. This strategy has enabled progress toward standardized first-in-human trials, underscoring the scalability of cocktail-based products.

In clinical practice, many programs now converge on a hybrid strategy that integrates both approaches [143]. A defined cocktail is initially used to ensure coverage in the face of strain diversity, followed by longitudinal retesting of respiratory isolates and adaptation of phage composition as dominant lineages or resistance profiles shift [143]. This mixed model is already reflected in the CF compassionate use experience, where 6 of 9 patients received cocktails, and 3 received monophage therapy, all within a controlled total-dose cap of 1 × 10^10^ PFU per administration [142]. From a translational perspective, this framework allows investigators to balance individualized potency with manufacturable consistency, while explicitly addressing resistance dynamics and within-host evolution in chronic pulmonary infection. Table 2 highlights the major preclinical and clinical studies of phage therapy against lung infections.

## 8. Regulatory, Manufacturing, and Safety Considerations

Bacteriophage therapies in the United States are regulated as biological drugs by the FDA’s Center for Biologics Evaluation and Research (CBER). As of 2025, no phage-based therapeutics have received formal FDA approval for clinical use; all such therapies remain experimental and are accessible only through investigational pathways via Investigational New Drug (IND) applications, which require extensive data on manufacturing processes and preclinical safety [147]. Although multiple clinical trials are currently underway, no definitive efficacy has yet been demonstrated in Phase III trials [148]. The FDA has acknowledged the potential for accessing phage therapy through compassionate use programs; however, it has not issued specific regulatory guidance tailored to phage-based therapeutics. Inhaled phage products must demonstrate safety, purity, and potency through comprehensive clinical trials before licensure [149]. Similarly, the European Medicines Agency (EMA) has not approved any bacteriophage therapies in Europe, although regulatory frameworks are being developed to support their integration into the medicinal product pipeline [150]. Within the European Union, therapeutic phages are classified as biological medicinal products and therefore require full marketing authorization [151].

Recent regulatory developments include the European Pharmacopoeia’s adoption of standardized quality criteria for human phage therapy products and the publication of a draft guideline on the quality aspects of phage therapy products for public consultation. Both the EMA and national regulatory authorities recognize the need for regulatory flexibility to accommodate the evolving nature of phage therapies, which may require modifications to maintain efficacy against emerging bacterial resistance [151]. Several European countries have implemented magistral pharmacy regulations that allow the provision of tailored phage therapies, exemplified by Belgium’s initiative permitting custom formulations without formal marketing authorization [148].

The World Health Organization (WHO) has identified bacteriophages as a potential strategy to combat antimicrobial resistance; however, phage therapy remains largely unapproved in many countries [152]. In Eastern Europe, phage therapies have been used for decades, often outside established regulatory frameworks [148]. WHO Europe is actively facilitating scientific and regulatory discussions to strengthen the evidence base for phage therapy, to develop guidance to support broader adoption once safety and efficacy are adequately demonstrated. Overall, the regulatory framework for inhaled phage therapy is still evolving, underscoring the need for robust clinical trials to transition phage therapies from experimental interventions to formally recognized treatments.

Modern cocktail development is shifting from broadening by addition to rational design based on receptor complementarity, quantitative interaction testing (phage and phage–antibiotic), and systematic matching of candidate phages to patient isolates. However, standardization remains constrained because each phage’s host range and apparent efficacy are context dependent, and multi-phage interactions can be complex enough that “more phages” is not. The EMA draft guideline puts this reality into practice by requiring justification for combining phage active substances, discussion of potential interactions, justification of limits on the maximum number combined, and, in use, stability, including compatibility studies when mixing at administration.

The most important technical challenge is measurement. The EMA draft states that, for multi-phage products, potency should be determined for each component unless otherwise justified, using separate plaque-based assays with bacterial strains not susceptible to other phages in the combination, an expectation that becomes increasingly difficult and sometimes biologically awkward as cocktails grow, target polymicrobial infections, or include phages with overlapping functions. In practice, poor standardization can result in hidden failures where the label indicates a cocktail composition, but one component has degraded or is subpotent, undermining efficacy and potentially accelerating resistance evolution against remaining active phages; conversely, overly broad cocktails can increase manufacturing complexity, cross-contamination risk, or immunological exposure without providing proportional benefit [153]. Best practice solutions emerging in the literature and regulatory direction include defining a target product profile tied to a clinically realistic bacterial panel; formalizing susceptibility testing workflows in clinical microbiology laboratories to reduce protocol heterogeneity; using design heuristics such as non-redundant receptor targeting and interaction screening; and implementing standardized potency determination, including automation, high-throughput approaches [154].

The most advanced approach to variability control treats phage products as biotechnology-derived biologics. This strategy defines explicit critical quality attributes (CQAs) and links them to critical process parameters (CPPs). It is supported by a seed lot system, process validation, expanded process analytical technologies (PATs), and single-use manufacturing. These measures aim to improve robustness and reduce contamination and drift [153]. Nonetheless, phage preparations are unusually vulnerable to many sources of stacked variability. Sources include biological variability in host bacterial physiology and lysis kinetics; physical instability, aggregation, adsorption to surfaces, and shear sensitivity affecting infective titer; genetic changes during propagation, which require explicit monitoring of genomic stability; and analytical variability in plaque-based assays. The outcomes of these assays depend strongly on the bacterial strain and culture conditions [155]. The practical safety and efficacy implications are obvious. Batch variability can shift the effective dose, alter the relative composition of a cocktail, or change impurity burdens (e.g., pyrogens, residual DNA/proteins). This can result in inconsistent clinical response, unexpected inflammatory reactions, or misinterpretation of clinical trial endpoints and PK/PD relationships [153]. Proposed best practices, in line with ICH quality frameworks and the EMA draft, include a QbD-based control strategy (ICH Q11) with predefined acceptance criteria in specifications (ICH Q6B). Other practices include comparability exercises for process changes (ICH Q5E), explicit hold-time studies, and the incorporation of sequencing-based genetic stability checks as an in-process or campaign-level control. These ensure the manufactured active substance remains representative of the master seed [156].

Current GMP-focused manufacturing of therapeutic phages follows a distinct biologic’s framework. This includes regulated upstream propagation in a specified bacterial production strain and downstream purification to eliminate host-derived impurities and contaminants while maintaining infectivity; the process ends with formulation and fill/finish. Throughout, route-specific microbiological quality controls are applied. Both the EMA draft and Ph. Eur. 5.31 emphasize the necessity of a characterized bacterial cell bank and a phage seed lot system, and highlight the need to prevent cross-contamination between phages and hosts. They also require pertinent release and shelf-life specifications, including obligatory tests for identity, potency, and microbiological quality [155]. The main scientific and manufacturing challenge is the extraction of phage drug substances from bacterial lysates, which are rich in pyrogens and other bacterial components. Purification must eliminate endotoxins, pyrogens, host cell proteins, host cell DNA, and induced prophage contaminants. However, these steps can reduce recovery rates or favor specific subpopulations, especially for giant or structurally fragile phages. Additionally, managing multi-phage portfolios carries a higher risk of mix-ups and cross-contamination, making proper facility structuring and documentation crucial for segregation and traceability [155].

EU GMP expectations pertinent to numerous phage products include Annex 1 for sterile manufacturing (fully applicable since August 2024) and Annex 2 for biologically active substances. These, in conjunction with the EMA phage guideline, require that developers substantiate their sterility strategy for sterile versus non-sterile PTMPs with specified microbiological quality, demonstrate impurity clearance for pyrogens and bacterial-derived impurities, and validate manufacturing and holding durations under regulated storage conditions [157]. In the United States, the general cGMP requirements for finished pharmaceuticals and biological products (e.g., 21 CFR Parts 211 and 600) establish foundational standards. At the same time, expectations for endotoxin and pyrogen control are delineated in FDA guidance on sampling plans, retesting, and method selection for bacterial endotoxin testing, particularly pertinent for phage products derived from Gram-negative lysates [158].

Safety and efficacy in manufacturing require more than scale-up. Poor impurity or microbiological control can lead to adverse inflammatory events. Excessive purification can lower the infectious titer or stability, which may make a product seem ineffective [153]. Emerging best practices include biologics-style lifecycle validation, regulatory-ready documentation, and risk-based impurity control. They also stress the need for pyrogen control in line with Ph. Eur. principles and for investment in scalable purification operations. For example, chromatography workflows can achieve multi-log reductions in endotoxin levels and ensure high phage recovery rates [159]. Key deficiencies remain. These include: (i) the lack of standardized, route-specific impurity criteria for phage products, especially for inhaled or topical uses where traditional endotoxin standards may not fit; (ii) no validated rapid microbiological techniques and real-time clearance monitoring for phage matrices; and (iii) no regulatory-grade Good Manufacturing Practice (GMP) frameworks for small batch or patient-specific production that guarantee safety without limiting access [155].

The European standards now state explicit requirements for genomic safety. Ph. Eur. 5.31 requires that bacterial production strains have defined antibiotic susceptibility profiles and documented chromosome plasmid sequences. Host strains and phage seed lots must lack sequences for harmful factors, such as antibiotic resistance genes, toxins, and prophage modules, unless justified and authorized. The EMA draft guideline also calls for comprehensive genome sequencing and annotation for master phage seed lots. They also require sequence-based characterization and genome analysis for harmful sequences in cell banks and phage seed lots [155]. The EMA insists on evaluating transducing capacity and gene checklists. Even strictly lytic phages can cause gene transfer through generalized transduction, so excluding temperate phages is not enough. This poses a challenge. Standardized and validated transduction risk assays are less advanced than sequencing; annotation remains uncertain for many phage genes, many of which are unknown or poorly annotated. Poor genomic screening threatens both safety and efficacy. Toxin genes or resistance markers might be introduced, or regulatory confidence may drop due to inconsistencies over time [159]. Best practice now means using tiered controls: (i) whole genome sequencing with curated analysis for unwanted genetic markers; (ii) phenotypic confirmation of lytic behavior when needed; (iii) genetic stability monitoring through sequencing; and (iv) transparent reporting of genome maps and annotation assumptions to aid regulatory review [155]. Critical policy and research gaps remain. Developers and regulators lack reference databases, consensus screening protocols for unknown phage ORFs, defined thresholds for acceptable genomic drift, and common tools for consistent assessment of transduction risk [159].

Inhalation delivery introduces additional formulation challenges, as phages must remain effective in both liquid and dry powder forms [105]. These formulations must preserve phage infectivity throughout manufacturing, storage, and administration, requiring careful management of shear stress during nebulization and protection against desiccation in dry powder inhalers, often through the use of stabilizing excipients [105]. Particle size distribution is critical for effective lung deposition, and comprehensive stability studies in accordance with International Council for Harmonization (ICH) guidelines are required to confirm long-term maintenance of titer and purity [130]. Until recently, the absence of standardized pharmacopeial monographs for phage products compelled manufacturers to rely on proprietary QC methods. The introduction of a dedicated European Pharmacopoeia chapter is expected to establish a foundational framework for quality requirements, enabling clearer benchmarks and harmonized testing approaches essential for regulatory approval of inhaled phage therapies. Effective QC remains fundamental to ensuring the safety, potency, and consistency of these complex biological products administered to sensitive pulmonary tissue [151].

Bacteriophage therapy has evolved within two primary regulatory paradigms: a standardized product model and a personalized “precision medicine” model. The standardized model uses pre-manufactured phage products, such as defined phage cocktails, that undergo conventional drug-approval processes. While this approach ensures consistent quality and safety, it is constrained by regulatory limitations due to its inflexibility. Modifications to phage compositions, often necessary to address bacterial resistance, require extensive regulatory review. Existing frameworks, including European Directive 2001/83/EC, do not readily accommodate such adaptability, creating tension between the dynamic nature of phage therapy and prolonged approval timelines. Consequently, European regulatory bodies are exploring adaptive licensing mechanisms to enable more rapid updates to phage formulations [151]. In contrast, the personalized phage therapy model offers greater flexibility by enabling the preparation of customized phage cocktails tailored to individual patients, typically in hospital or pharmacy settings rather than in large-scale manufacturing facilities [160]. This approach diverges from traditional pharmaceutical regulation, as each preparation is unique and not intended for widespread commercial distribution. Several countries, including Belgium, permit pharmacies to compound personalized phage therapies under specific regulatory exemptions, improving patient access while simultaneously introducing challenges related to regulatory oversight, clinical evidence generation, and reimbursement [161].

Regulatory authorities are increasingly examining hybrid approaches that integrate both models. Initiatives such as the Transatlantic Task Force on Antimicrobial Resistance have proposed frameworks designed to balance flexibility with safety, including the use of pre-approved phage libraries and the introduction of new regulatory categories such as “adaptive biologics” [160]. Currently, personalized phage therapies are primarily confined to clinical research settings or compassionate use programs, particularly in the United States, where they are often classified as experimental single-patient interventions under FDA regulations [162]. Overall, the regulatory landscape reflects an ongoing effort to reconcile the reliability of standardized models with the adaptability of personalized therapies, aiming to ensure patient safety while enabling individualized treatment options [148].

Therapeutic phages generally exhibit a favorable safety profile compared with conventional antibiotics, as lytic phages selectively infect bacterial cells without targeting human tissues, thereby minimizing cytotoxicity and systemic adverse effects [163,164]. Nevertheless, safety considerations remain, particularly concerning immunogenicity and endotoxin contamination, which are especially relevant for inhaled phage therapies. Immunogenic responses may occur when phage components activate the host immune system, leading to the release of pro-inflammatory cytokines and potential inflammatory reactions [165]. Reported adverse effects are typically mild and may include fever or bronchospasm, particularly at higher doses or with less-purified formulations [166]. Additionally, adaptive immune responses can lead to the production of neutralizing antibodies against phages, potentially reducing therapeutic efficacy over repeated administrations [52]. Endotoxin contamination originating from Gram-negative bacterial hosts presents a significant safety risk, as endotoxins can induce severe inflammatory responses, fever, and hypoxia. Regulatory authorities, therefore, impose strict endotoxin limits on phage products to ensure patient safety [167]. To meet these requirements, manufacturers employ advanced purification strategies and conduct routine endotoxin testing to verify compliance. While residual risks persist, clinical studies have reported endotoxin levels within acceptable limits when rigorous purification and quality controls are applied [168].

All in all, the safety of inhaled phage therapy depends heavily on effective management of immune responses and strict control of endotoxin contamination through robust manufacturing and quality assurance practices. Early clinical data suggest that, when appropriate safeguards are implemented, phages can be administered safely with minimal toxicity. Continued advancement of inhaled phage therapies will require comprehensive safety evaluations, with particular emphasis on immunogenicity and endotoxin profiling, to support broader clinical adoption.

## 9. Challenges and Future Directions

Despite the promising potential application of bacteriophages as therapeutic agents for pulmonary infections, multiple biological, technological, regulatory, and clinical challenges remain to be addressed before phage therapy can be fully integrated into respiratory therapy [45]. The complex lung microenvironment, host immune interactions, limitations in large-scale manufacturing, and delivery limitations collectively shape both current obstacles and future opportunities [169]. Overcoming these challenges will be critical to realizing the full therapeutic potential of bacteriophages in respiratory medicine.

One of the major challenges associated with phage-based therapeutics is the development of bacterial resistance to phages. In the lung microenvironment, high bacterial densities, biofilm formation, and heterogeneous oxygen and nutrient gradients amplify this challenge [170,171]. Pathogens such as *P. aeruginosa* and *Acinetobacter baumannii* can rapidly acquire different resistance mechanisms, including receptor modification, restriction-modification enzymes, and abortive infection pathways [172]. Moreover, in chronic respiratory conditions such as CF and bronchiectasis, persistent bacterial colonization creates prolonged selective pressure that may facilitate the accelerated emergence of phage resistance [126]. However, phage resistance does not necessarily lead to treatment failure, as it is often accompanied by fitness trade-offs that can reduce bacterial virulence or restore susceptibility to antibiotics. Future therapeutic strategies are expected to emphasize the rational formulation of phage cocktails that target multiple bacterial receptors, periodic phage rotation, and the incorporation of phages that exploit conserved bacterial structures less prone to mutation [141]. Understanding phage–bacteria co-evolution within the lung ecosystem remains a critical research priority.

The pulmonary immune system presents both an opportunity and a barrier to effective phage therapy. Following inhalation, bacteriophages encounter multiple immune components, including alveolar macrophages, neutrophils, complement proteins, and mucosal antibodies, all of which can contribute to rapid phage clearance [105,173]. Innate immune recognition may limit phage persistence and bioavailability, particularly during repeated administration. In addition, adaptive immune responses, including the generation of neutralizing antibodies, may further reduce therapeutic efficacy over time [44,174].

At the same time, phages have been shown to exert immunomodulatory effects, including anti-inflammatory actions that may be beneficial in hyperinflammatory lung conditions [175]. Balancing immune activation and tolerance will be essential for safe and effective therapy. Although phages are generally species-specific, off-target effects on commensal bacteria could disrupt microbial homeostasis and the resident lung microbiota, with potential consequences for lung health and immune regulation [176]. Future research should emphasize longitudinal studies assessing immune responses, microbiota dynamics, and long-term safety following inhaled phage administration.

The successful translation of phage therapy into clinical practice is also a major challenge, requiring robust, scalable, and standardized manufacturing processes that comply with GMP guidelines. Phages are biologically complex products, and batch-to-batch variability, contamination with bacterial endotoxins, and stability issues remain significant obstacles [177]. Unlike conventional antibiotics, phage manufacturing relies on bacterial host systems, which introduces additional biosafety and QC concerns. Establishing GMP-compliant phage production pipelines will require standardized bacterial host strains, validated purification protocols, and comprehensive characterization of phage identity, potency, and purity [178,179]. Advances in upstream fermentation technologies, downstream purification, such as chromatography and ultrafiltration, and formulation science are expected to improve scalability and regulatory acceptance [180].

Efficient and reproducible delivery of phages to the lower respiratory tract remains a major technical challenge. Aerosolization exposes phages to mechanical stresses, including shear forces, dehydration, and temperature fluctuations, which can reduce viability [181,182]. Moreover, achieving uniform deposition across diseased lung regions, particularly in patients with mucus obstruction or altered airway architecture, is difficult [183]. Precise dosage control is further complicated by variability in inhalation patterns, nebulizer performance, and patient adherence [184]. Future directions include developing phage-friendly nebulization systems, dry-powder inhalers, and protective formulations incorporating stabilizing excipients. Nanocarrier-based delivery systems may enhance phage stability, prolong lung residence time, and improve penetration into biofilms [105,185]. Establishing standardized dosing regimens and pharmacokinetics-pharmacodynamics models for inhaled phages remains a critical unmet need.

Combining therapies offers a promising approach to overcoming several limitations of phage monotherapy. Phage-antibiotic combinations have demonstrated synergistic effects, including enhanced bacterial killing, reduced emergence of resistance, and restoration of antibiotic susceptibility [42,186]. In pulmonary infections caused by MDR pathogens, such combinations may provide an effective alternative to last-line antibiotics.

Phage-nanoparticle systems represent an emerging frontier, enabling targeted delivery, controlled release, and protection from immune clearance. Nanoparticles can be engineered to co-deliver phages with antibiotics, anti-inflammatory agents, or quorum-sensing inhibitors, thereby addressing multiple pathogenic mechanisms simultaneously [173]. While preclinical data are encouraging, comprehensive safety evaluations and translational studies are required before clinical implementation.

Artificial intelligence (AI) and machine-learning (ML) approaches are poised to revolutionize phage therapy by enabling rapid phage selection, optimization, and personalization. AI-driven platforms can analyze bacterial genomic data to predict phage susceptibility, identify optimal phage combinations, and anticipate the development of resistance [187,188]. In the context of pulmonary infections, such tools could support personalized treatment strategies tailored to individual patients’ pathogens, lung microbiota, and disease phenotypes [189]. However, the successful adoption of AI-driven approaches will depend on the availability of high-quality datasets, transparent algorithms, and regulatory acceptance of computational decision-support tools.

For phage therapy to be successfully integrated into routine pulmonary care, clear clinical guidelines, regulatory pathways, and education initiatives are required. Phage therapy should be positioned as a complementary tool within antibiotic stewardship programs, particularly for the management of MDR infections [117]. Defining appropriate indications, treatment algorithms, and outcome measures will be essential to ensure rational and evidence-based application. Clinician familiarity, patient acceptance, and logistical considerations such as rapid access to phage libraries will significantly influence real-world implementation. Collaborative networks linking clinicians, microbiologists, and phage production centers may facilitate timely and effective treatment decisions [190].

Looking forward, advances in synthetic biology, formulation technologies, and systems medicine are expected to refine phage design and delivery. Well-designed randomized clinical trials, supported by standardized manufacturing and regulatory frameworks, will ultimately determine the role of bacteriophages as mainstream therapeutic agents for pulmonary infections. With continued innovation and strategic integration, phage therapy holds the potential to become a critical component of future respiratory infection management in the era of escalating antimicrobial resistance.

## 10. Conclusions

Over the past decade, bacteriophage therapy has advanced from a largely experimental concept to a scientifically grounded antimicrobial strategy with growing relevance for pulmonary infections. Major progress has been made in elucidating phage–bacterial host interactions in the respiratory tract, engineering lytic phages with improved stability and host range, and developing sophisticated pulmonary delivery platforms, including optimized nebulization systems, dry powder inhalers, and encapsulated formulations. Preclinical models now provide strong evidence of efficacy against MDR pathogens, biofilm-associated infections, and chronic lung disease. At the same time, early clinical experiences, particularly in CF and VAP, demonstrate biological activity, acceptable safety, and therapeutic potential.

Despite these advances, important challenges continue to limit widespread clinical implementation. These include variability in phage pharmacokinetics, incomplete understanding of immune interactions, the risk of resistance emergence, and the need for standardized criteria for potency, purity, and stability. From a translational perspective, regulatory fragmentation remains a major barrier, as existing frameworks were designed for fixed chemical drugs rather than adaptive, biologically replicating agents. Harmonized international regulatory pathways are urgently needed to address phage characterization, manufacturing quality systems, personalized formulations, and post-administration monitoring. In parallel, industrial scalability must be addressed through the development of robust, GMP-compliant production platforms, validated purification pipelines, scalable formulation technologies, and sustainable phage banks that support both off-the-shelf products and patient-specific therapies.

Successful integration of bacteriophage therapy into respiratory medicine will depend heavily on interdisciplinary collaboration. Progress at the clinical level requires close coordination among microbiologists, pulmonary physicians, pharmacists, immunologists, and bioengineers. At the same time, effective translation further depends on regulatory scientists, manufacturing specialists, and data scientists to establish standardized workflows, rapid susceptibility testing, and adaptive treatment algorithms. Such cross-sector collaboration is essential to bridge laboratory innovation, clinical feasibility, and industrial execution.

Looking forward, bacteriophage therapy holds strong potential to become an integral component of mainstream antimicrobial practice. Rather than replacing antibiotics, phages are likely to be incorporated into multimodal treatment strategies that include antibiotics, biologics, and advanced drug-delivery systems. With continued technological refinement, regulatory alignment, and clinical validation, inhaled phage therapeutics could transition from compassionate-use interventions to standardized, evidence-based treatments, offering a durable and adaptable solution to the escalating crisis of drug-resistant pulmonary infections.

## Figures and Tables

**Table 1:** Features of conventional antibiotics and bacteriophages in the treatment of pulmonary infection diseases.

Features	Conventional Antibiotics	Bacteriophages	Reference
Primary mechanism of action	Chemically inhibit essential bacterial processes, e.g., cell wall synthesis, protein synthesis, and DNA replication.	Infect bacteria directly, hijack their replication machinery, and induce bacterial lysis.	[42,78]
Target specificity	Broad-spectrum activity; often affects commensal lung and gut microbiota.	Highly specific to target bacterial strains, maintaining normal microbiota.	[42,45,79,80,81]
Activity against biofilms	Poor penetration, reduced efficacy.	May disrupt biofilms in some settings, particularly when depolymerase enzymes are present; however, efficacy depends strongly on phage characteristics, EPS composition, and biofilm maturity.	[67]
Pharmacokinetics in lung tissue	Limited penetration in mucus-rich environments.	It can diffuse through mucus layers, improving control in focal pulmonary niches, but mucus viscosity, charge interactions, EPS trapping, and immune clearance modulate diffusion.	[47,82]
Resistance dynamics	Resistance is increasingly prevalent; mutations can preserve or enhance fitness within the treated niche.	Resistance may emerge via modification of EPS remodeling receptors, but is often transient due to phage–bacteria co-evolution.	[43,78,83]
Effect on microbiota	Broadly disrupts the gut microbiome and selects for resistant flora with repeated use.	Minimal disruption due to high host specificity, though a narrow host range may limit coverage of diverse or evolving pathogen populations	[42,43,44]
Self-amplification at the site of infection	No, self-amplification requires repeated high-dose administration.	Yes, phages replicate locally in the presence of susceptible bacteria, although amplification is constrained by biofilm structure, metabolic heterogeneity, and immune clearance.	[42,43,44]
Adaptability	Fixed activity profile; Cannot adapt to changing infection dynamics.	Highly adaptable; Easily customized for resistance emergence.	[24,44,45]
Safety profile	Risk of toxicity, allergic reactions, and drug–drug interactions.	Well tolerated with low systemic toxicity.	[42,43,45,67]

**Table 2:** Major preclinical and clinical studies of phage therapy for lung infections.

Study Type	Pathogen	Model or Population	Route	Phage Format	Key Quantitative Details	Ref.
Preclinical PK/PD	*P. aeruginosa*	BALB/c mouse pulmonary PK and lung infection PD model	Intratracheal pulmonary delivery	Single phage (PEV31)	PK: 10^7^ vs. 10^9^ PFU intratracheal; mice n = 4/timepoint; sampled 0, 1, 2, 4, 8, 24 h. Lung half-life ~8 h for both doses. PD: 10^9^ PFU given 2 h after MDR inoculation; lung phage increased ~2 log_10_ by 16 h in the presence of bacteria; phage-resistant isolates seen. Linking pulmonary exposure to bacterial presence.	[120]
Preclinical dose-response	*P. aeruginosa*	Neutropenic BALB/c murine lung infection	Intratracheal (pulmonary)	Single phage (PEV31), 3 dose levels	Infection: 2 × 10^4^ CFU intratracheal. Treatment at 2 h post-inoculation with 7.5 × 10^4^ PFU, 5 × 10^6^ PFU, or 5 × 10^8^ PFU; PBS control. Necropsy at 2 h and 24 h. Lung bacterial load reduced by 1.3–1.9 log_10_ at 24 h across doses; resistance emergence increased with higher dose. Bacterial reduction was not strongly dose-dependent over this range, supporting careful dose selection.	[121]
Preclinical VAP combination (adjunctive)	*P. aeruginosa* (PAO1)	Murine VAP with ventilator-induced lung injury	Infection: intratracheal; Treatment: intraperitoneal	Cocktail (2 phages, equal proportion) ± meropenem	Infection: 5 × 10^4^ CFU/20 µL intratracheal after 4 h mechanical ventilation. Treatment at 4 h and 16 h post-infection: meropenem 10 mg/mouse/injection, or phage cocktail ~5 × 10^7^ PFU per phage per injection, or combination. The timing and endpoints mirror those of an ICU-relevant VAP design.	[127]
Preclinical MRSA (rat)	MRSA (*S. aureus* AW7)	Mechanically ventilated Wistar rat MRSA pneumonia	Aerosolized (vibrating mesh nebulizer) and/or IV	4-phage cocktail (2003, 2002, 3A, phage K) ± linezolid	Inoculum: ~1 × 10^10^ CFU via endotracheal tube after 4 h ventilation. Phage cocktail at 1.5 × 10^10^ PFU/mL; aerosol treatment volume 2 mL, ~10 min per dose (particle size ~3.1 µm). Dosing schedule: phages at 2, 12, 24, 48, 72 h; primary endpoint survival at 96 h. Aerophages alone, 50% survival; IV phages, 50%; combined aerophage + IV phage, 91% (higher than monotherapy). Comparing inhaled vs. systemic vs. combined phage delivery, with survival as a hard endpoint.	[144]
Compassionate use (expanded access)	MDR *P. aeruginosa*	Single patient, chronic MDR lung infection (Kartagener syndrome)	Nebulized aerosol	Personalized monophage (vFB297)	Dosing: phage resuspended in 5 mL PBS, nebulized over 20 min; daily dose 5 × 10^9^ PFU for 5 consecutive days, then 2 additional doses 2 days later. Samples were collected before each dose and 7–8 h after each administration. Clinical improvement was reported with reduced bacterial load and evidence of phage replication in sputum. Only still single-patient evidence.	[83,129]
Compassionate cohort (CF)	MDR or PDR *P. aeruginosa* in CF	9 adults with CF (compassionate basis)	Nebulized (jet nebulizer)	Personalized: cocktail (2–3 phages) or monophage	Regimen: inhaled phage twice daily (inpatients) or daily (outpatients) for 7–10 days; total dose 1 × 10^10^ PFU. Microbiology: sputum PsA decreased from a median 2.6 × 10^8^ CFU/mL pre-therapy to a median 2.6 × 10^4^ CFU/mL post-therapy (5–18 days after; median reduction 10^4^ CFU/mL). Lung function: ppFEV1 improved by 8% at 21–35 days. The pulmonary endpoints for compassionate use (quantitative sputum CFU plus ppFEV1 change), for both monophage and cocktail use.	[130]
Clinical trial (CF)	*P. aeruginosa* in CF	Randomized, placebo-controlled trial protocol (CYPHY, NCT04684641)	Inhaled nebulization	Algorithm-guided single phage selection from the YPT-01 library	Investigational product: phages provided as 1 mL solution of a single phage at standard dose ≥ 1 × 10^8^ phage particles; minimum acceptable dose 1 × 10^8^ PFU/mL; placebo is PBS + 10 mM MgSO_4_.	[145]
Clinical case report with a biodistribution focus	XDR *A. baumannii*	Elderly female; inhaled phage therapy in 2 phases	Inhaled	Personalized phage	Phage DNA was detected in blood only during the first 4 days of the second phase (Ct (cycle threshold) 32.6–35.3). Sputum phage Ct decreased from ~45 to 14.7 during the first phase, then stabilized at ~28.5–26.8 in the second phase. Fecal Ct decreased (35.5 → 22.5, 32.6 → 22.7), suggesting intestinal accumulation during inhaled therapy. Systemic exposure and off-target distribution after inhaled dosing, but the study reports qPCR signals rather than infectious PFU counts.	[146]

## Data Availability

No new data were created or analyzed in this study.
